# Nursing Students' Perspectives on Integrating Artificial Intelligence Into Clinical Practice and Training: A Qualitative Descriptive Study

**DOI:** 10.1002/hsr2.70728

**Published:** 2025-04-21

**Authors:** Moustaq Karim Khan Rony, Sumon Ahmad, Dipak Chandra Das, Sabren Mukta Tanha, Tuli Rani Deb, Mosammat Ruma Akter, Mst. Amena Khatun, Md Ibrahim Khalil, Umme Rabeya Peu, Mst. Rina Parvin, Daifallah M. Alrazeeni, Fazila Akter

**Affiliations:** ^1^ Miyan Research Institute International University of Business Agriculture and Technology Dhaka Bangladesh; ^2^ Leading University Sylhet Bangladesh; ^3^ Shanto‐Mariam University of Creative Technology Dhaka Bangladesh; ^4^ Nursing Service Bangabandhu Sheikh Mujib Medical University Dhaka Bangladesh; ^5^ National Institute of Advanced Nursing Education and Research Dhaka Bangladesh; ^6^ Pundra University Science and Technology Bogura Bangladesh; ^7^ Institute of Social Welfare and Research University of Dhaka Dhaka Bangladesh; ^8^ Chattogram Imperial College of Nursing Chattogram Bangladesh; ^9^ Bangladesh Army (AFNS Officer), Combined Military Hospital Dhaka Bangladesh; ^10^ Department Prince Sultan Bin Abdul Aziz College for Emergency Medical Services King Saud University Riyadh Saudi Arabia; ^11^ Department of Health and Functioning Western Norway University of Applied Sciences Bergen Norway

**Keywords:** artificial intelligence, clinical practice, ethical considerations, healthcare technology, nursing education, student perspectives

## Abstract

**Background:**

The integration of artificial intelligence (AI) into healthcare has introduced transformative tools to enhance clinical decision‐making and streamline workflows. In nursing, a profession characterized by human‐centric care, AI adoption offers both significant opportunities and notable challenges. However, the perspectives of nursing students, future professionals, on integrating AI into clinical practice and education remain underexplored.

**Aim:**

This study aimed to explore nursing students' perceptions of incorporating AI into their clinical training and professional practice, with a focus on identifying benefits, challenges, and potential areas for improvement.

**Methods:**

A qualitative descriptive design explored the experiences and attitudes of 25 nursing students from five colleges in Dhaka, Bangladesh. Participants were purposively sampled to ensure diverse educational and clinical backgrounds. Semi‐structured interviews in Bangla, lasting 40–50 min, were audio‐recorded, transcribed, and translated into English. Data were collected from May 8, 2024 to August 10, 2024. Data were analyzed using thematic analysis to identify patterns and themes. Credibility was ensured through member checking, dependability via an audit trail, and confirmability through peer debriefing. Data visualization tools were used to map thematic relationships effectively.

**Results:**

Thematic analysis revealed four major themes: (1) AI integration in nursing education, (2) ethical and professional concerns, (3) preparedness for AI‐driven practice, and (4) AI's impact on nursing practice. Participants expressed both optimism about AI's potential to improve accuracy and efficiency and apprehension about their readiness to use AI effectively in practice.

**Conclusion:**

The findings underscore the need for comprehensive curriculum reforms that incorporate AI training, address ethical concerns, and emphasize the role of AI as a supportive tool rather than a replacement for human expertise. These insights provide a roadmap for integrating AI into nursing education while preserving the compassionate core of nursing practice.

## 1 Introduction

The rise of artificial intelligence (AI) in healthcare has sparked transformative changes, introducing innovative tools that improve patient care, support clinical decision‐making, and streamline operational processes [1, 2]. In nursing, a field grounded in holistic, human‐centered care, the integration of AI presents both exciting opportunities and complex challenges [3]. Emerging technologies like predictive analytics, AI‐assisted diagnostics, and automated documentation systems have the potential to enhance nursing practice by freeing professionals to focus more on patient care and less on administrative tasks [4, 5]. However, the adoption of AI in nursing is not without complications, particularly for nursing students who are currently preparing to enter the profession [6]. As the future of healthcare, their perspectives on incorporating AI into clinical practice and education remain underexplored [7].

Nursing students hold a critical role in shaping the future of healthcare, and their attitudes toward AI adoption can provide valuable insights into its possibilities and limitations [8]. Positioned as both learners and aspiring professionals, they offer a unique perspective that highlights not only the readiness of nursing education to embrace technological advancements but also the challenges they anticipate in clinical settings [9, 10]. While much of the existing research on AI in healthcare focuses on its applications in medicine and allied health fields, nursing often receives less attention [11]. Yet, nursing is distinct in its emphasis on blending technical expertise with ethical responsibility and interpersonal care, factors that heavily influence how AI technologies are perceived and utilized in practice [12]. Although there is limited research specifically examining nursing students' perspectives on AI, a few recent studies have started to address this gap. For instance, recent investigations have explored nursing students' knowledge, attitudes, and anxiety regarding AI applications, as well as their preparedness and concerns about integrating AI into clinical care [13]. These studies highlight the urgency of understanding how future nurses perceive AI, especially in resource‐limited settings where infrastructure and policy support may be lacking [14]. By examining the views of nursing students, we can shed light on key issues such as the ethical implications of AI, its potential to alter the nurse–patient dynamic, and whether current training adequately prepares students for AI‐integrated healthcare [15, 16].

The integration of AI into nursing education requires a fundamental shift in traditional teaching approaches [17]. Nursing curricula must not only cover technical competencies but also foster critical thinking about the ethical, legal, and professional aspects of AI [18]. For students, adapting to these changes may evoke a range of responses, from excitement about the efficiencies AI can bring to skepticism about its impact on human‐centered care [19]. Additionally, challenges like unequal access to AI‐enabled tools during training and varying levels of digital literacy among students should be addressed [20, 21]. Understanding these complexities is essential to designing educational frameworks that empower nursing students with the confidence and skills to navigate an increasingly technology‐driven healthcare system [22].

In Bangladesh, nursing education is overseen by the Bangladesh Nursing and Midwifery Council (BNMC), with most programs focused on foundational clinical competencies, community health, and patient‐centered care. However, formal inclusion of AI‐related content in nursing curricula is still largely absent [23]. Exposure to digital health tools varies significantly between institutions, with limited infrastructure and training resources acting as major barriers to technological integration [24]. At the clinical level, the application of AI in healthcare is still emerging, with limited but growing adoption, primarily in private healthcare institutions [25]. AI is gradually being introduced through initiatives such as diagnostic imaging tools, hospital information systems, and patient data management platforms [26]. However, widespread integration remains constrained due to infrastructure limitations, lack of formal training programs, and minimal policy‐level emphasis [27].

This lack of structured exposure makes it essential to explore how nursing students understand and perceive AI, particularly as they prepare to enter an evolving healthcare landscape where digital technologies will play an increasingly central role. Therefore, this study aimed to explore the perceptions of nursing students regarding the integration of AI into clinical practice and training. By examining their experiences, attitudes, and concerns, the research identified the barriers and opportunities associated with AI adoption in nursing.

## 2 Methods

### 2.1 Study Design

This study used a qualitative descriptive approach to explore nursing students' views on incorporating artificial intelligence (AI) into clinical practice and education. By following the guidelines outlined in the Consolidated Criteria for Reporting Qualitative Research (COREQ) checklist [28], the study upheld a transparent and thorough methodological structure. This approach was chosen for its ability to capture detailed and meaningful insights into the participants' attitudes, experiences, and expectations. It enabled the researchers to delve into the students' distinct perspectives within the broader scope of nursing education and practice, providing a deeper understanding of how AI could be integrated into their learning and clinical experiences.

### 2.2 Study Setting

The research took place in Dhaka, the bustling capital of Bangladesh, known for its rich cultural diversity and a variety of nursing institutions with different educational frameworks. Dhaka was selected because it includes both public and private nursing colleges, offering a well‐rounded representation of institutional diversity and student demographics. Using purposive sampling, five nursing colleges in the city were chosen for the study. The selection criteria included accessibility, a diverse student population, and the presence of clinical training programs in their curricula. All the nursing education facilities involved in this study are accredited by the Bangladesh Nursing Council, ensuring standardized educational quality and regulatory oversight. These factors ensured that the participants represented a broad range of educational and practical experiences, enhancing the richness of the study's findings.

### 2.3 Participant Recruitment

Participants in this study were chosen through purposive sampling, ensuring the selection of individuals best equipped to provide meaningful perspectives on incorporating artificial intelligence (AI) into clinical practice and training. Eligibility requirements included active enrollment in undergraduate or postgraduate nursing programs, current participation in clinical training, and a willingness to contribute to the research. While prior direct experience with AI tools was not mandatory, several participants had encountered or observed AI applications during their clinical rotations, which contributed to their ability to reflect on its integration and potential. Students without clinical experience or those who declined to participate were excluded. A total of 25 nursing students were interviewed (Table 1), with the sample size guided by the principle of data saturation—ensuring that all key themes were thoroughly explored and no new insights emerged from additional interviews.

**Table 1:** Participants' demographics.

ID	Age	Gender	Course of enrollment	Current semester	Clinical experience (years)	College type
R1	22	Female	MSN	4th	4	Public
R2	20	Male	BSc	8th	2	Private
R3	29	Male	MSN	2nd	9	Private
R4	27	Female	Post‐basic	2nd	6	Private
R5	26	Female	MSN	3rd	5	Public
R6	22	Female	BSc	7th	3	Private
R7	20	Male	BSc	8th	2	Public
R8	27	Female	Post‐basic	4th	9	Public
R9	28	Female	MSN	3rd	9	Public
R10	20	Male	BSc	6th	2	Private
R11	23	Female	Post‐basic	2nd	5	Private
R12	20	Male	BSc	6th	2	Public
R13	26	Female	Post‐basic	1st	8	Private
R14	25	Male	Post‐basic	1st	2	Public
R15	21	Male	BSc	5th	1	Private
R16	23	Female	BSc	4th	3	Private
R17	24	Male	Post‐basic	3rd	6	Public
R18	22	Male	BSc	7th	3	Private
R19	26	Female	Post‐basic	4th	8	Private
R20	23	Male	BSc	6th	2	Public
R21	21	Male	BSc	8th	1	Private
R22	24	Female	Post‐basic	4th	6	Private
R23	25	Female	MSN	1st	7	Private
R24	21	Female	BSc	3rd	3	Private
R25	22	Male	BSc	3rd	2	Public

*Note:* The Master of Science in Nursing (MSN) is a 2‐year program with 4 semesters, while the Bachelor of Science in Nursing (BSN) spans 4 years with 8 semesters. The Post‐Basic nursing program, designed for those with a 3‐year diploma in nursing, is a 2‐year course comprising 4 semesters.

### 2.4 Data Collection

Data were collected from May 8, 2024 to August 10, 2024. Semi‐structured interviews were conducted to gain in‐depth insights into nursing students' perspectives on integrating artificial intelligence (AI) into clinical practice and education. The interview schedule was developed through a thorough review of existing literature and consultations with nursing educators and healthcare professionals. Participants were asked the following questions: “How do you perceive the role of artificial intelligence in improving patient care within clinical practice?” “What opportunities do you think AI can create for nursing professionals in clinical settings?” “What challenges do you anticipate when integrating AI into your clinical training and future nursing practice?” “What specific training or resources do you think nursing students need to effectively use AI in their practice?” “How comfortable are you with the idea of incorporating AI technologies into your daily nursing responsibilities?” and “What role do you think nursing educators should play in preparing students for the integration of AI into healthcare systems?”. These questions were carefully refined for cultural and contextual relevance, incorporating feedback from a panel of experts, which included two senior nursing educators with over 10 years of academic and clinical teaching experience, one health informatics specialist, and one AI researcher with experience in healthcare technology implementation. Face‐to‐face interviews were held in a guest room setting at the respective colleges, creating a relaxed and private atmosphere that encouraged open and honest discussions. Each interview, conducted in Bangla to facilitate natural expression, lasted around 40–50 min. The audio recordings were transcribed verbatim and later translated into English by a bilingual professional, ensuring both accuracy and the preservation of nuanced responses.

### 2.5 Ethical Considerations

The study followed strict ethical guidelines and received approval from the Institutional Ethics Review Board of Imperial College of Nursing (Study/HR/NUR08032024). Participants were given a clear and comprehensive explanation of the study's objectives, methods, potential challenges, and anticipated benefits to ensure transparency and understanding. Before participating, each individual provided written informed consent, confirming their voluntary agreement to take part in the research. Pseudonyms were assigned to protect anonymity, and all data were securely stored on encrypted devices to ensure confidentiality. Participation was fully voluntary, with participants clearly informed of their right to withdraw at any time without any repercussions. These measures were carefully implemented to uphold ethical standards and prioritize the participants' rights, privacy, and overall well‐being throughout the research process.

### 2.6 Data Analysis

The data were analyzed using thematic analysis, adhering to the systematic approach described by Nowell et al. [29]. This approach was chosen for its flexibility and systematic framework, which effectively identifies, analyzes, and reports themes within qualitative data [30]. The process followed six stages: becoming familiar with the data, creating initial codes, identifying themes, reviewing themes, defining and naming themes, and drafting the final report [29]. Audio recordings of the interviews were transcribed verbatim in Bangla and later translated into English to preserve the depth and nuances of the data. Three researchers immersed themselves in the transcripts through repeated readings, manually coding meaningful segments related to participants' perceptions, challenges, and suggestions regarding AI. The codes were subsequently organized into potential themes that reflected broader patterns within the data set. These themes were meticulously reviewed and refined to ensure they authentically represented the participants' perspectives. Additionally, Python programming was utilized for its robust data organization and visualization capabilities. Tools like Matplotlib were used to create a thematic tree, providing a clear and systematic categorization and visualization of the identified themes.

### 2.7 Rigor

To ensure the rigor of the study's findings, several strategies were employed at different stages of the research, incorporating diverse levels of researcher involvement. Credibility was ensured during the analysis and interpretation process by involving three researchers in member checking [31]. This involved sharing preliminary findings with a subset of participants to confirm the accuracy of the interpretations. Dependability was maintained by keeping a detailed audit trail that documented all decisions and processes throughout data collection and analysis [32], a task primarily managed by two additional researchers. Moreover, confirmability was strengthened through peer debriefing sessions [33] involving the principal author, senior authors, and research advisors, which aimed to minimize personal bias and ensure the findings were firmly grounded in the data. Furthermore, Transferability was strengthened by including comprehensive, detailed descriptions of the study's context and participant demographics in the reporting phase [34], allowing readers to evaluate the findings' relevance and applicability to other settings. By combining these strategies and fostering collaborative researcher involvement, the study reinforced the credibility, reliability, and applicability of its outcomes.

## 3 Results

### 3.1 Theme: AI Integration in Nursing Education

The incorporation of AI into nursing education is transforming the field, equipping nursing students with sophisticated tools to support more accurate and informed decision‐making. Participants highlighted AI's potential to serve as a “second brain,” capable of identifying patterns and generating insights that might be missed in high‐pressure scenarios (Table 2). However, this promise is often hindered by a lack of adequate training and support. Many nursing students feel overwhelmed, describing the learning process as akin to “osmosis” or trying to navigate a map without clear directions.

**Table 2:** Themes and descriptions based on participants' quotes.

Theme	Subtheme	Description	Example quotes	Keywords
AI integration in nursing education	Enhancing clinical decision‐making	Describes how AI acts as an indispensable tool in identifying patterns, ensuring safety, and augmenting the decision‐making process during high‐pressure scenarios. It provides critical support but still relies on human interpretation for context.	“AI feels like having a second brain that never tires, helping us notice patterns we might otherwise miss on a long shift.” (R4) “When time is short and the stakes are high, having AI to back up our decisions feels like a safety net we didn't know we needed.” (R13) “It's amazing how AI can sift through so much data… but at the end of the day, it still needs us to give it meaning and context.” (R19)	Clinical decision‐making, patterns, safety net, support, data analysis, human–AI collaboration
	Challenges in AI training	Highlights the frustrations and obstacles nursing students face due to inadequate training, lack of hands‐on experience, and unclear guidance, leaving them unprepared for AI integration.	“Sometimes it feels like they want us to learn AI by osmosis, like we're supposed to just magically understand it. Umm, that's really frustrating!” (R8) “We're being told how important AI is, but without proper guidance, it's like being handed a map with no directions.” (R12) “Without proper hands‐on experience, learning AI feels like trying to swim without water.” (R23)	AI training, frustration, lack of resources, inadequate guidance, hands‐on learning
Ethical and professional concerns	Patient privacy issues	Raises concerns about the security, confidentiality, and ethical implications of using AI systems, especially regarding sensitive patient information and trust in AI technology.	“How much of our patients' lives we're feeding into systems that might not be secure is deeply unsettling to think about.” (R25) “I can't help but wonder if we don't fully trust the AI with sensitive data, how can we expect our patients to?” (R2)	Patient privacy, data security, ethical concerns, confidentiality, trust in AI
	Nurse–patient relationships	Emphasizes the importance of maintaining human connection and emotional support in patient care, aspects that AI cannot replicate, ensuring holistic and empathetic care remains central.	“No matter how advanced AI becomes, it can't hold a patient's hand or comfort them during a tough moment.” (R17) “Patients come to us for care, not calculations. They need to see a human, not just a screen. In my opinion, this human connection is what defines true healing.” (R7)	Human connection, empathy, emotional support, holistic care, patient trust
	Accountability for AI decisions	Explores the tension and anxiety among nurses about being held accountable for AI‐driven errors, despite following guidelines, creating stress about trust and responsibility.	“Knowing we could be blamed for a mistake made by AI, even when we followed all the guidelines, is nerve‐wracking.” (R18) “There's this constant worry about what happens when an AI recommendation goes wrong. Do we trust it, or double‐check everything?” (R5) “Sometimes, it feels like we're walking a tightrope between trusting technology and taking full responsibility for its outcomes.” (R11)	Accountability, trust in AI, responsibility, decision‐making stress, AI errors
Preparedness for AI‐driven practice	Readiness for AI tools	Illustrates the apprehension and lack of confidence nursing students feel regarding the use of AI in clinical settings, stemming from inadequate preparation and training.	“I love the idea of AI making our work easier, but right now, I don't feel ready to rely on it in real‐life situations.” (R15) “AI is being presented as this amazing thing, but without proper training, it's just another stressor for us.” (R3) “Every time I hear about a new AI tool, I feel more behind—it's like we're expected to keep up without being taught how.” (R20)	Readiness, training, confidence gap, stress, preparation, real‐life scenarios
	Curriculum gaps	Points out significant gaps in nursing education, where AI integration is minimal or theoretical, lacking practical, hands‐on training that could build confidence and competence in AI use.	“Our classes prepare us for everything from patient care to emergencies, but there's barely any talk about how to work with AI.” (R24) “If AI is going to be part of our future, why isn't it already a bigger part of our education? It feels like we're being set up to catch up later.” (R1) “We need more than just theory; hands‐on practice with AI tools would make a huge difference in our confidence and competence.” (R9)	Curriculum, education gaps, hands‐on training, competence, theoretical focus, preparation
AI's impact on nursing practice	Improving accuracy and efficiency	AI's quick and reliable processing capabilities provide an additional layer of safety by reducing human errors and enhancing efficiency in critical moments, offering invaluable support to nursing staff.	“The way AI processes information so quickly is mind‐blowing, it's like having a supersmart colleague who never takes a break.” (R16) “It's comforting to know AI can catch errors we might miss when we're exhausted or under pressure. I think it gives us an extra layer of security in critical moments.” (R22)	Accuracy, efficiency, error prevention, support, critical moments, information processing
	Limitations in human‐centric care	Acknowledges the irreplaceable value of emotional intelligence and human connection in care, which AI, despite its advances, cannot replicate or fully understand.	“AI can't read emotions or provide comfort; it doesn't know what it means to care for a patient who's scared or in pain.” (R3) “No matter how much AI improves, there's something irreplaceable about a nurse's intuition and emotional connection with a patient. I believe this is what truly defines quality care.” (R10)	Emotions, empathy, human‐centric care, emotional intelligence, intuition, quality care
	Balancing AI with human judgment	Highlights the need for a collaborative approach where AI enhances but does not overshadow human expertise, ensuring nursing decisions retain their empathetic and intuitive core.	“Achieving balance means using AI as support without allowing it to overshadow our expertise and instincts. Of course, that's often easier said than done.” (R6) “I see AI as a tool, not a replacement—it can guide us, but it can't replace the human touch.” (R14) “We need to work alongside AI, not depend on it completely; it's about keeping the heart in healthcare.” (R17) “Trusting AI is important, but trusting ourselves to make the final call is even more critical.” (R21)	Balance, human judgment, collaboration, trust, expertise, human intuition, support

#### 3.1.1 Subtheme: Enhancing Clinical Decision‐Making

AI tools can process vast amounts of data with remarkable speed, offering critical insights that elevate decision‐making in clinical environments. Participants likened these tools to tireless assistants that boost both accuracy and efficiency. However, they underscored that AI cannot replace the nuanced judgment of nursing students, which requires contextualizing and interpreting data to align with the complex realities of patient care.

AI feels like having a second brain that never tires, helping us notice patterns we might otherwise miss on a long shift.

(R4)

When time is short and the stakes are high, having AI to back up our decisions feels like a safety net we didn't know we needed.

(R13)

It's amazing how AI can sift through so much data… but at the end of the day, it still needs us to give it meaning and context.

(R19)

#### 3.1.2 Subtheme: Challenges in AI Training

A major concern expressed by participants is the lack of sufficient training in using AI tools. Many nursing students shared feelings of frustration over the absence of hands‐on experiences, which leaves them unprepared to fully adopt these technologies. Some compared the experience to being handed “a map with no directions,” emphasizing the need for structured and practical guidance. One participant was referring to her educators and academic institution when expressing these frustrations. She perceived that the responsibility to provide clear guidance and practical AI exposure lay primarily with her nursing education program.

Sometimes it feels like they want us to learn AI by osmosis, like we're supposed to just magically understand it. Umm, that's really frustrating!

(R8)

We're being told how important AI is, but without proper guidance, it's like being handed a map with no directions.

(R12)

Without proper hands‐on experience, learning AI feels like trying to swim without water.

(R23)

### 3.2 Theme: Ethical and Professional Concerns

Ethical and professional concerns surrounding AI use in nursing are critically important, especially in areas like patient privacy, trust, and accountability. Participants expressed significant apprehensions about the security of sensitive patient data, questioning whether AI systems can truly safeguard this information. Another key concern is the potential impact of technology on the nurse‐patient relationship, with participants warning that over‐reliance on AI risks weakening this vital human connection (Table 3).

**Table 3:** Challenges and recommendations with respondent references.

Identified challenges	Recommendations	Suggested implementation	Respondents' ID
Lack of understanding of AI concepts	Develop foundational AI training	Incorporate beginner‐friendly AI modules into the curriculum to address gaps in knowledge	R8, R12
Limited hands‐on experience with AI tools	Offer practical AI training	Use simulations and workshops to provide real‐world applications of AI in nursing	R23, R9
Concerns over patient privacy and data security	Strengthen data security protocols	Ensure AI tools meet stringent data protection standards and educate nurses on privacy practices	R25, R2
Fear of losing human connection in patient care	Emphasize empathy alongside AI	Include training on balancing technical tools with emotional intelligence in caregiving	R17, R7
Accountability for AI‐related errors	Define shared accountability frameworks	Develop policies clearly outlining roles and responsibilities when using AI systems	R18, R5, R11
Curriculum gaps in AI education	Expand AI integration in nursing curricula	Introduce structured AI modules with theoretical and practical components tailored for nursing students	R24, R1
Feeling unprepared for AI integration in clinical practice	Gradual integration of AI in practice	Start with simpler AI tools in training and progressively increase complexity to build competence	R15, R3
Over‐reliance on AI tools	Promote critical thinking and human judgment	Provide training that emphasizes the nurse's role in interpreting AI outputs while maintaining clinical expertise	R6, R14, R21
Limitations in addressing emotional and human aspects	Highlight human‐centric roles	Focus on AI as a support system rather than a replacement, preserving the nurse–patient relationship	R3, R10
Balancing AI efficiency with human intuition	Foster collaborative AI‐human decision‐making	Teach strategies for integrating AI insights with clinical experience to achieve optimal outcomes	R6, R17

#### 3.2.1 Subtheme: Patient Privacy Issues

Protecting patient privacy emerged as one of the most pressing concerns among nursing students. Many participants expressed unease about the potential for sensitive data to be mishandled or misused by AI systems, leading to a lack of trust in the technology. This mistrust can hinder the adoption of AI in healthcare. Participants underscored the significance of adopting robust data security measures to safeguard patient information and foster trust among nursing students and patients alike.

How much of our patients' lives we're feeding into systems that might not be secure is deeply unsettling to think about.

(R25)

I can't help but wonder if we don't fully trust the AI with sensitive data, how can we expect our patients to?

(R2)

#### 3.2.2 Subtheme: Nurse–Patient Relationships

While AI can enhance efficiency and support decision‐making, it cannot replace the emotional connection and empathy that student nurses bring to patient care. Participants stressed that patients often turn to student nurses for comfort and human interaction during vulnerable moments—something no technology can replicate. They cautioned against over‐relying on AI, which could diminish this essential aspect of nursing care. Maintaining the human connection is seen as vital to preserving the heart of nursing practice.

No matter how advanced AI becomes, it can't hold a patient's hand or comfort them during a tough moment.

(R17)

Patients come to us for care, not calculations. They need to see a human, not just a screen. In my opinion, this human connection is what defines true healing.

(R7)

#### 3.2.3 Subtheme: Accountability for AI Decisions

Participants voiced concerns about the lack of clarity around accountability when AI systems make errors. Nursing students are wary of being held responsible for mistakes made by AI, even when they have followed all established guidelines. This uncertainty creates a dilemma: should they trust AI recommendations or verify every outcome manually? To resolve this, participants emphasized the need for shared accountability frameworks that clearly define roles and responsibilities, ensuring that nursing students are not unfairly blamed for errors caused by AI systems.

Knowing we could be blamed for a mistake made by AI, even when we followed all the guidelines, is nerve‐wracking.

(R18)

There's this constant worry about what happens when an AI recommendation goes wrong. Do we trust it, or double‐check everything?

(R5)

Sometimes, it feels like we're walking a tightrope between trusting technology and taking full responsibility for its outcomes.

(R11)

### 3.3 Theme: Preparedness for AI‐Driven Practice

The rapid advancement of AI tools has left many nursing students feeling unprepared for their integration into clinical practice. Participants pointed to a significant gap between the pace of technological innovation and the level of educational preparedness in nursing programs. Without comprehensive training, AI becomes an additional stressor rather than a helpful resource. To bridge this divide, participants emphasized the need to rethink nursing curricula and incorporate practical, hands‐on experiences with AI tools to build confidence and readiness for real‐world applications.

#### 3.3.1 Subtheme: Readiness for AI Tools

In this study, participants expressed discomfort and uncertainty about their ability to effectively use AI tools. A lack of proper training has left some viewing AI as more of a challenge than an asset. This sentiment underscores the importance of creating skill‐based training programs that focus on real‐world applications, ensuring nursing students feel confident and equipped to use AI in their practice.

I love the idea of AI making our work easier, but right now, I don't feel ready to rely on it in real‐life situations.

(R15)

AI is being presented as this amazing thing, but without proper training, it's just another stressor for us.

(R3)

Every time I hear about a new AI tool, I feel more behind—it's like we're expected to keep up without being taught how.

(R20)

#### 3.3.2 Subtheme: Curriculum Gaps

A significant barrier to AI preparedness is the lack of AI‐focused content in nursing curricula. Participants pointed out that most programs provide little to no training on working with AI, leaving nursing students to “catch up” later in their careers. They emphasized the need for comprehensive education that goes beyond theoretical knowledge to include hands‐on practice. Updating curricula to include practical AI training is seen as essential for preparing nursing students to thrive in an AI‐driven healthcare environment.

Our classes prepare us for everything from patient care to emergencies, but there's barely any talk about how to work with AI.

(R24)

If AI is going to be part of our future, why isn't it already a bigger part of our education? It feels like we're being set up to catch up later.

(R1)

We need more than just theory; hands‐on practice with AI tools would make a huge difference in our confidence and competence.

(R9)

### 3.4 Theme: AI's Impact on Nursing Practice

AI is revolutionizing nursing practice by improving accuracy and efficiency, while also encouraging consideration of its limitations in providing human‐centered care. Participants acknowledged the value of AI as a supportive tool but stressed the need to maintain a balance between technology and human judgment. This balance ensures that while AI delivers its benefits, it does not compromise compassionate, patient‐centered care.

#### 3.4.1 Subtheme: Improving Accuracy and Efficiency

Participants highly valued AI's ability to process data with speed and precision. It provides an extra layer of safety, especially in high‐pressure scenarios, acting as a dependable, tireless colleague. However, they emphasized that the true potential of AI is realized only when paired with the expertise and intuition of nursing students, who can interpret its outputs within the nuanced context of patient care.

The way AI processes information so quickly is mind‐blowing, it's like having a supersmart colleague who never takes a break.

(R16)

It's comforting to know AI can catch errors we might miss when we're exhausted or under pressure. I think it gives us an extra layer of security in critical moments.

(R22)

#### 3.4.2 Subtheme: Limitations in Human‐Centric Care

While AI enhances operational efficiency, it falls short in areas that require empathy and emotional intelligence. Participants stressed that quality care is rooted in the human connection nursing students build with their patients, particularly during moments of fear or vulnerability. True healing, they asserted, is defined by the emotional and intuitive interactions that AI cannot replicate.

AI can't read emotions or provide comfort; it doesn't know what it means to care for a patient who's scared or in pain.

(R3)

No matter how much AI improves, there's something irreplaceable about a nurse's intuition and emotional connection with a patient. I believe this is what truly defines quality care.

(R10)

#### 3.4.3 Subtheme: Balancing AI With Human Judgment

Participants highlighted the need for a careful balance between leveraging AI's capabilities and relying on human expertise. AI should be viewed as a supportive tool, not a substitute for the critical thinking and instincts of nursing students. This balance ensures that healthcare remains efficient while retaining its human touch, preserving the compassion and intuition that define nursing care.

Achieving balance means using AI as support without allowing it to overshadow our expertise and instincts. Of course, that's often easier said than done.

(R6)

I see AI as a tool, not a replacement—it can guide us, but it can't replace the human touch.

(R14)

We need to work alongside AI, not depend on it completely; it's about keeping the heart in healthcare.

(R17)

Trusting AI is important, but trusting ourselves to make the final call is even more critical.

(R21)

## 4 Discussion

**Figure 1:**
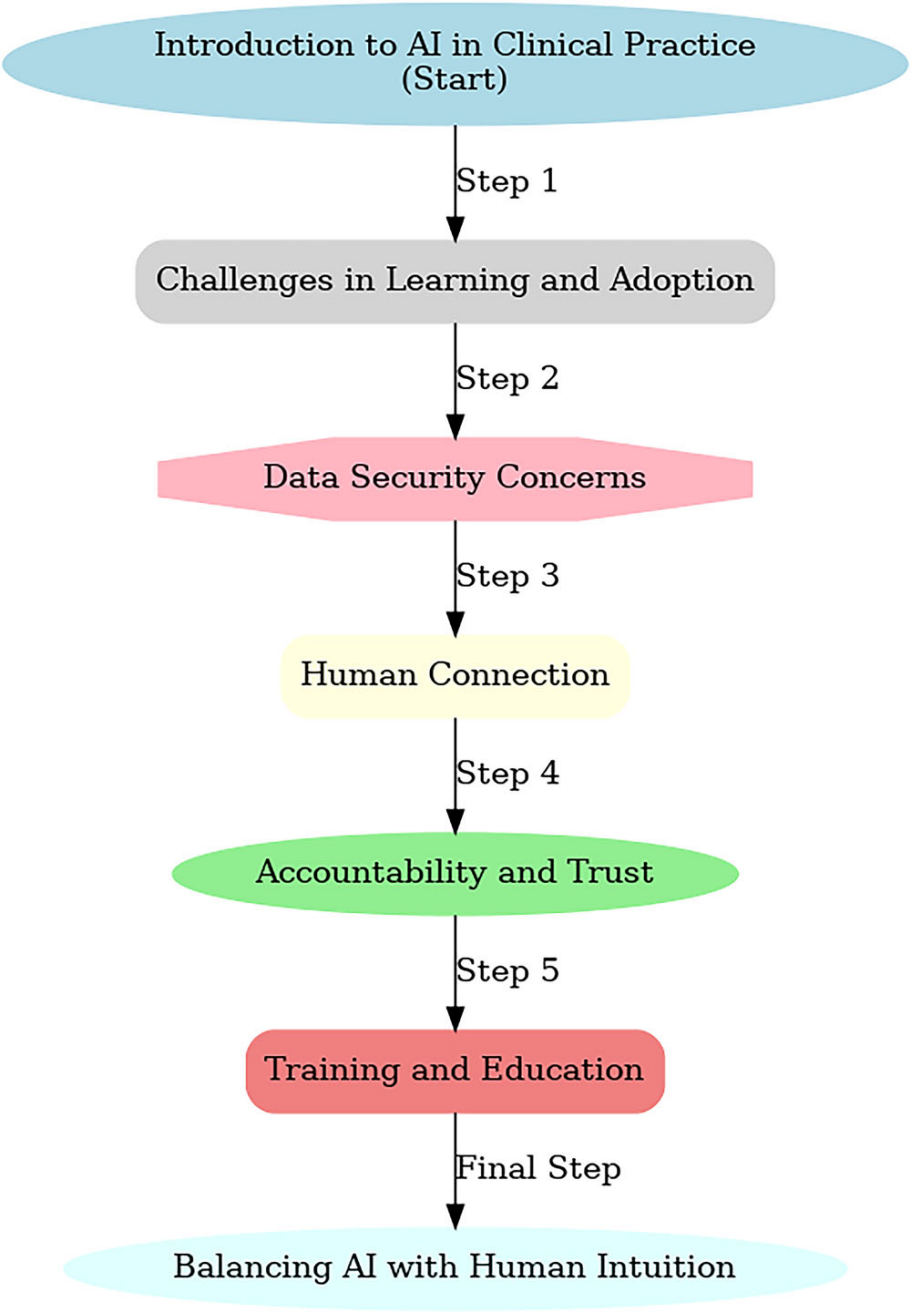
Flowchart of AI integration in clinical practice. This flowchart, developed from participants' insights and thematic analysis, outlines key steps for integrating AI into nursing clinical practice. It highlights challenges in adoption, data security, the importance of human connection, accountability, and the need for structured training—culminating in the balance between AI and human intuition.

This study examined nursing students' views on integrating artificial intelligence (AI) into clinical practice and training, revealing a mix of potential benefits and challenges. While AI integration into nursing education appears promising, the research also highlighted notable gaps in both preparedness and curriculum design. One significant advantage of AI was its ability to support clinical decision‐making, particularly through analyzing complex data sets and identifying patterns that improve patient care. These findings align with existing research, such as that of Ronquillo et al., which emphasized AI's role in enhancing diagnostic accuracy [35]. Similarly, studies by Makhlouf et al. demonstrated that AI minimized medication errors, adding a critical layer of safety in clinical settings [36]. However, this contrasts with outcomes in regions that have sophisticated AI educational frameworks but still highlight the absence of practical training as a significant challenge [37, 38]. This underscores the need for nursing education to incorporate practical, AI‐focused training programs (Figure 1).

In addition to gaps in training, concerns have also been raised in the broader literature about the potential for AI to contribute to the deskilling of healthcare professionals or reduce opportunities for critical thinking. As AI systems increasingly take on clinical decision‐support roles, there is a risk that over‐reliance may lead nursing students and practitioners to depend too heavily on technology, potentially weakening their clinical judgment and problem‐solving skills [39]. These considerations further support the need for curricula that emphasize critical engagement with AI tools, rather than passive use [40].

Ethical and professional concerns emerged as another crucial area of focus. Concerns about patient privacy and data security were consistent with global studies, such as those by Khosravi et al., which noted healthcare professionals' apprehension about the confidentiality risks posed by AI‐driven systems [41]. Additionally, the study highlighted the delicate balance between harnessing AI's efficiency and preserving the core of human‐centered care. Watson's theory of human caring emphasized the irreplaceable value of empathy and emotional presence in nursing, which AI alone cannot replicate [42]. Findings from Ruksakulpiwat et al. further supported this, showing that patients prioritize emotional and relational support, raising concerns that over‐reliance on AI could diminish trust in healthcare [43]. These insights stress the importance of developing ethical guidelines and training programs that integrate AI without undermining humanistic values in nursing care.

The study also brought attention to the preparedness of nursing students for AI‐driven practice, revealing a critical disconnect between AI's rapid advancement and current educational curricula. These findings echo sentiments shared by Ng et al., who pointed out the lag in nursing programs' adoption of digital health tools [44]. Similarly, research by Oduoye et al. showed that inadequate exposure to AI in nursing education led to increased stress and reduced confidence among students [45]. By contrast, institutions in technologically advanced countries showcased the benefits of comprehensive AI education [46, 47]. For example, simulation‐based training programs in some European nations have been effective in building students' confidence and competence in using AI tools [48, 49]. Adopting such strategies on a global scale could help address the readiness gap.

AI's impact on nursing practice highlighted its transformative potential alongside its limitations. The technology's ability to improve accuracy and efficiency in clinical settings aligns with research such as Rony et al., which showed how AI reduced diagnostic errors and enhanced care coordination [50]. Bohn and Anselmann also illustrated how AI's capacity to process large data sets facilitated faster decision‐making during emergencies [51]. Moreover, findings from Gulirmak Guler and Sen Atasayar [52] emphasized the critical role of emotional intelligence in patient interactions, a domain where AI falls short. These observations underscore the need to view AI as a complementary tool rather than a replacement, ensuring that human judgment and intuition remain central to nursing practice.

Striking the right balance between AI and human expertise emerged as a nuanced challenge. The study highlighted nursing students' psychological struggle to trust AI while maintaining accountability for care decisions. Literature such as Raymond et al. advocated for a collaborative model where AI complements human intuition [53]. Likewise, studies by Hamedani et al. explored how interdisciplinary collaboration between AI developers and healthcare professionals can build trust and foster successful AI implementation in clinical settings [54]. Encouraging teamwork and establishing clear accountability frameworks could help alleviate concerns about liability while promoting a balanced and effective approach to AI integration [55, 56].

Importantly, this study identified a set of practical recommendations (Table 3) that could help mitigate the barriers to AI integration in nursing. Participants proposed foundational training to address the lack of understanding of AI concepts, and recommended simulation‐based learning to build hands‐on experience. Ethical concerns, such as data privacy, were met with suggestions to implement stringent data protection protocols and define shared accountability frameworks for AI‐related decisions. Moreover, to preserve humanistic values, respondents advocated for training that emphasizes empathy and relational care alongside technological competence. These recommendations not only align with broader literature but also offer context‐specific strategies for improving AI readiness among nursing students. By embedding these solutions into nursing education and policy frameworks, institutions can create an environment where AI complements rather than compromises care delivery.

## 5 Insights From the Study

This study highlights the transformative role artificial intelligence (AI) can play in shaping the future of nursing education and practice. With its ability to enhance decision‐making, streamline workflows, and improve care precision, AI is positioned as a crucial tool in the evolving healthcare landscape. Participants in the study described AI as a “second brain,” offering valuable support in high‐pressure situations where rapid and accurate decisions are essential. However, the findings emphasize that integrating AI successfully requires more than just technological innovation. Key issues, such as the lack of structured training, ethical concerns around patient data privacy, and fears of over‐reliance on technology, must be addressed. Importantly, the study underscores that while AI is a powerful resource, it should enhance rather than replace the compassion and intuition that are central to nursing. This insight provides a blueprint for integrating AI in ways that strengthen both technical proficiency and human‐centered care.

## 6 Notable Advantages and Drawbacks of the Study

The study has several notable strengths that contribute to its credibility and reliability. Its qualitative design enabled an in‐depth exploration of nursing students' lived experiences and perspectives, offering detailed insights into their views on AI integration. By selecting diverse nursing colleges in Dhaka, the researchers captured a wide range of educational and clinical experiences, ensuring a varied and representative sample. The use of rigorous methodologies, such as member checking, peer debriefing, and thematic analysis, further strengthened the trustworthiness of the findings. Additionally, the inclusion of culturally relevant interview protocols provided context‐specific data that reflected the local healthcare environment.

Nonetheless, the study has some limitations. Its geographic focus on Dhaka restricts the generalizability of its findings to other regions or countries, particularly those with different healthcare systems or educational frameworks. Cultural and contextual factors unique to Bangladesh may also influence perceptions of AI, making it challenging to apply these insights universally. Furthermore, the reliance on self‐reported data introduces potential biases, as participants may have presented overly favorable or cautious views. Another potential limitation is the variability in participants' clinical experience and stages of education, which, although intentional to ensure diversity, may have influenced the consistency of responses and introduced a degree of variability in perceptions. While this heterogeneity was purposefully sought to enhance the study's transferability, it also presents a limitation, as the diversity in educational and clinical backgrounds may have diluted or skewed some of the thematic findings. These limitations highlight the need for additional research in other settings to validate and expand upon these findings.

## 7 Directions for Future Research

Future research should broaden the scope of AI integration in nursing to include diverse cultural, geographical, and institutional contexts. By examining various healthcare systems, researchers can develop a more comprehensive understanding of the challenges and opportunities AI presents. A key area of focus should be the development and evaluation of hands‐on, simulation‐based AI training programs to assess their effectiveness in preparing nursing students for real‐world applications. Longitudinal studies tracking the impact of AI‐centered curricula on clinical outcomes, patient satisfaction, and professional development over time would also provide valuable evidence for curriculum improvement.

Additionally, future studies should delve deeper into the ethical implications of AI in nursing. Exploring how AI affects trust, accountability, and interpersonal dynamics between nurses and patients can provide crucial insights into the psychological and relational aspects of technology adoption. Quantitative research could complement qualitative findings by measuring tangible benefits, such as improved workflow efficiency, reduced errors, and enhanced patient outcomes. A holistic approach to future research that considers the technical, ethical, and human dimensions of AI will be essential to guide its integration into nursing practice effectively.

## 8 Conclusions

This study underscores the significant potential of AI to transform nursing education and practice by enhancing decision‐making, improving accuracy, and streamlining processes. However, its successful integration requires careful planning and a commitment to addressing key challenges. Bridging curriculum gaps, addressing ethical concerns, and providing comprehensive training are crucial steps to ensure nursing students are well‐prepared to use AI effectively. Equally important is safeguarding the human connection that defines nursing, ensuring AI serves as a supportive tool rather than a substitute for human expertise. By prioritizing structured training, ethical safeguards, and a balanced approach to AI integration, nursing education and practice can evolve to harness the benefits of AI while maintaining compassionate care central to the profession.

## Author Contributions

**Moustaq Karim Khan Rony:** conceptualization, investigation, writing – original draft, visualization, validation, writing – review and editing, methodology, software, formal analysis, project administration, data curation, supervision, and resources. **Sumon Ahmad:** conceptualization, data curation, visualization, writing – original draft, and software. **Dipak Chandra Das:** conceptualization, methodology, formal analysis, writing – original draft. **Sabren Mukta Tanha:** investigation, data curation, methodology, writing – review and editing. **Tuli Rani Deb:** data curation, formal analysis, project administration, resources, writing – review and editing. **Mosammat Ruma Akter:** data curation, validation, writing – review and editing, project administration. **Mst. Amena Khatun:** formal analysis, investigation, visualization, writing – review and editing. **Md Ibrahim Khalil:** data curation, resources, project administration, writing – review and editing. **Umme Rabeya Peu:** formal analysis, validation, visualization, writing – review and editing. **Mst. Rina Parvin:** conceptualization, data curation, software, visualization, writing – review and editing. **Daifallah M. Alrazeeni:** supervision, validation, visualization, writing – review and editing. **Fazila Akter:** conceptualization, methodology, supervision, writing – original draft.

## Conflicts of Interest

The authors declare no conflicts of interest.

## Transparency Statement

The lead author Moustaq Karim Khan Rony affirms that this manuscript is an honest, accurate, and transparent account of the study being reported; that no important aspects of the study have been omitted; and that any discrepancies from the study as planned (and, if relevant, registered) have been explained.

## Data Availability

The data sets used and/or analyzed during the current study are available from the corresponding author upon reasonable request.
